# Encapsulation of Vitamins A and E as Spray-Dried Additives for the Feed Industry

**DOI:** 10.3390/molecules25061357

**Published:** 2020-03-17

**Authors:** Javiera Mujica-Álvarez, O. Gil-Castell, Pabla A. Barra, A. Ribes-Greus, Rubén Bustos, Mirko Faccini, Silvia Matiacevich

**Affiliations:** 1 Departamento de Ciencia y Tecnología de Alimentos, Facultad Tecnológica, Universidad de Santiago de Chile, Obispo Umaña 050, Estación Central, 9170201 Santiago, Chile; 2 Departamento de Ingeniería Química, Facultad de Ingeniería, Universidad de Santiago de Chile, Av. Libertador Bernardo O’Higgins 3363, Estación Central, 9170002 Santiago, Chile; ruben.bustos@usach.cl; 3 Instituto de Tecnología de Materiales, Universitat Politècnica de València, Camino de Vera s/n, 46022 València, Spain; ogilcastell@doctor.upv.es (O.G.-C.); aribes@ter.upv.es (A.R.-G.); 4 Departament d’Enginyeria Química, Escola Tècnica Superior d’Enginyeria, Universitat de València, Av. de la Universitat, s/n, 46100 Burjassot, Spain; 5 Centro de Excelencia en Nanotecnología (CEN), Leitat Chile, Calle Román Díaz 532, Providencia, Santiago 7500724, Chile; pbarra@leitat.cl (P.A.B.); mfaccini@leitat.org (M.F.); 6 Materials Chemistry Division, Leitat Technological Center, C/Pallars 179–185, 08005 Barcelona, Spain

**Keywords:** vitamin A, vitamin E, nanoemulsions, encapsulation, spray-drying

## Abstract

Encapsulated fat-soluble powders containing vitamin A (VA) and E (VE) were prepared as a feasible additive for extruded feed products. The effect of the encapsulating agents (Capsul-CAP^®^, sodium caseinate-SC) in combination with Tween 80 (TW) as an emulsifier and maltodextrin (MD) as a wall material on the physicochemical properties of emulsions and powders was evaluated. First, nanoemulsions containing MD:CAP:TW:VA/VE and MD:SC:TW:VA/VE were prepared and characterized. Then, powders were obtained by means of spray-drying and analyzed in terms of the product yield, encapsulation efficiency, moisture content, porosity, surface morphology, chemical structure, and thermal properties and thermo-oxidative/thermal stability. Results showed that although nanoemulsions were obtained for all the compositions, homogeneous microcapsules were found after the drying process. High product yield and encapsulation efficiency were obtained, and the presence of the vitamins was corroborated. The characteristics of the powders were mainly influenced by the encapsulating agent used and also by the type of vitamin. In general, the microcapsules remained thermally stable up to 170 °C and, therefore, the proposed encapsulation systems for vitamins A and E were suitable for the preparation of additives for the feed manufacturing through the extrusion process.

## 1. Introduction

Vitamins are organic compounds required for prosperous growth and health. They are often not synthesized by humans or animals and must be provided in the diet. Although vitamins can be classified according to several criteria, in terms of their solubility, they can be divided into water-soluble and fat-soluble. Fat-soluble vitamins include vitamins A (retinol, betacarotene), D (cholecalciferol), E (α-tocopherol), and K (phylloquinone). Among them, vitamins E and A receive great attention due to their nutritional values [1,2]. On the one hand, vitamin A contributes to form and to maintain healthy teeth, skeletal, skin and soft tissues [3]. On the other hand, vitamin E is an antioxidant that plays a key role in the immune system and metabolic processes [4]. However, it is still a challenge to combinate these vitamins with food due to their poor solubility water and relative instability during food processing and storage [2,5]. Therefore, they must be incorporated into hydrophilic matrices and protected.

Up to date, the challenge of the feed industry is to obtain stable vitamin-enriched powder microcapsules, that allow to decreasing their susceptibility to chemical degradation, especially at elevated temperatures, high oxygen levels, and light exposures [6,7,8,9,10,11,12,13,14,15,16]. Moreover, during extrusion, factors like the barrel and mass temperature, the screw rate, and throughput may affect to the retention and the destruction of the vitamins, and especially vitamin A and vitamin E [17]. In fact, the destruction of 90%–95% of both vitamins was observed in fish feed due to heat, the contact with the water, and during the stocking after the extrusion process [17].

The encapsulation based on nanoemulsions is a suitable technology, which may result in a stable powder after processing by means of the spray-drying technique. This technology is currently studied as a method to preserve nutrients over time, in addition to improving its stability, bioavailability, and release control of their active compounds [18,19,20,21,22,23]. The main encapsulated components are usually vitamins, flavors, or essential oils, among others, and may be protected from unfavorable environmental conditions ‒e.g., oxygen, light and heat‒ during handling and processing [16,22,24,25,26,27]. The success of encapsulation depends on the selection of appropriate encapsulating agent/surfactant and method. The most used encapsulating agents include carbohydrates such as polysaccharides ‒e.g., Arabic gum, maltodextrin and plain/modified starch‒, and proteins ‒e.g., gelatin, sodium caseinate‒. Carbohydrates are usually used in combination with other carbohydrates or proteins to provide higher functional properties. In this line, the sodium caseinate is widely used as a carrier for the encapsulation of lipophilic vitamins due to its intrinsic stability to form protein-ligand complexes and casein micelles [28,29]. Moreover, the amphiphilic nature and disordered structure of caseins promote a rapid adhesion to the surface of oil droplets to form a thick and entangled layer that protects the newly formed droplets against flocculation and coalescence [28,30]. On the other hand, maltodextrin has been employed as a stabilizer in emulsion formulations to bring more resistance to environmental stress [31,32,33]. However, the coverage of the oil droplet by sodium caseinate and maltodextrin may not be enough to permit the effective accumulation of the active compounds in the oil-water interface [31]. The use of Capsul^®^ may allow a complete coverage of oil droplets, and the incorporation of Tween80^®^ as emulsifier may increase the encapsulation efficiency, the droplet size, and therefore, the emulsion stability [8,15,16,18,26,34].

While the encapsulating method must be selected according to the physical and chemical properties of the core material and the encapsulating agents, the desirable size and shape of particles must be considered [2]. Indeed, for the feed industry, the powder form is commonly required. Although nano-spray-drying is useful to obtain nano-encapsulated compounds, its inherent high cost encouraged the optimization of the conventional spray-drying method to obtain microcapsules containing nanoparticles with low cost and high stability [22].

Therefore, the main objective of this study was to fabricate fat-soluble vitamin (A and E) powders by means of the combination of nanoemulsion and conventional spray-drying techniques. Moreover, the evaluation of the effect of the encapsulating agents on the physicochemical properties and thermal stability of the prepared powder microcapsules was proposed. Overall, this research provides valuable knowledge as a potential methodology to fabricate lipophilic vitamin-delivery systems for being used as additives for extruded feed products.

## 2. Results and Discussion

### 2.1. Emulsion Characterization

The particle size and the polydispersity index (PDI) are considered critical parameters of emulsions, related to their physical stability [21]. The emulsions with particle sizes around 100 nm are required due to their superior physical stability in comparison to conventional emulsions [8,18,21,35,36]. The PDI value was correlated to the spread of the particle size distribution and its values goes from 0 to 1. When the PDI value was close to 0, the physical stability of the emulsion was ensured, and was represented by a narrow size distribution [37,38,39]. Generally, values below 0.3 result in uniformity among the particle size distribution and good physical stability [26,40].

Table 1 gathers the Z-average size and the PDI for emulsions of both vitamins A (VA) and E (VE) with different combinations of encapsulating agents. Results showed that the procedure to obtain nanoemulsions was effective for all samples (≤100 nm). However, significant differences (*p* < 0.05) were obtained between vitamins. Emulsions of VE showed lower particle sizes (<80 nm) than those of VA (~100 nm), regardless of the encapsulating agent. The lowest particle size (75.1 nm) was obtained for the MD:CAP:TW:VE composition. According to the particle size, PDI values differed from one vitamin to the other. Emulsions of VE showed lower PDI (<0.28) than those of VA (>0.40). Indeed, emulsions of VE showed a monomodal size distribution whereas those of VA exhibited a broader size distribution. In particular, MD:CAP:TW:VA showed the highest PDI value, given the appearance of two different size populations, one at ~100 nm and a little one around 800 nm. Although the lowest particle size and PDI value suggested that VE nanoemulsions may be more stable, it is important to note that all the nanoemulsions showed good physical stability during 24 h of storage at 4 °C, and no destabilization mechanisms were observed until the drying process. Nevertheless, it is important to remark that the physical emulsion stability is not exclusively correlated to the particle size and distribution but also on other factors, such as the viscosity of the dispersed and continuous phases, the electrostatic and steric interaction between droplets, and the density of each phase, among others [21].

### 2.2. Powder Characterization

#### 2.2.1. Surface Morphology

**Figure 1:**
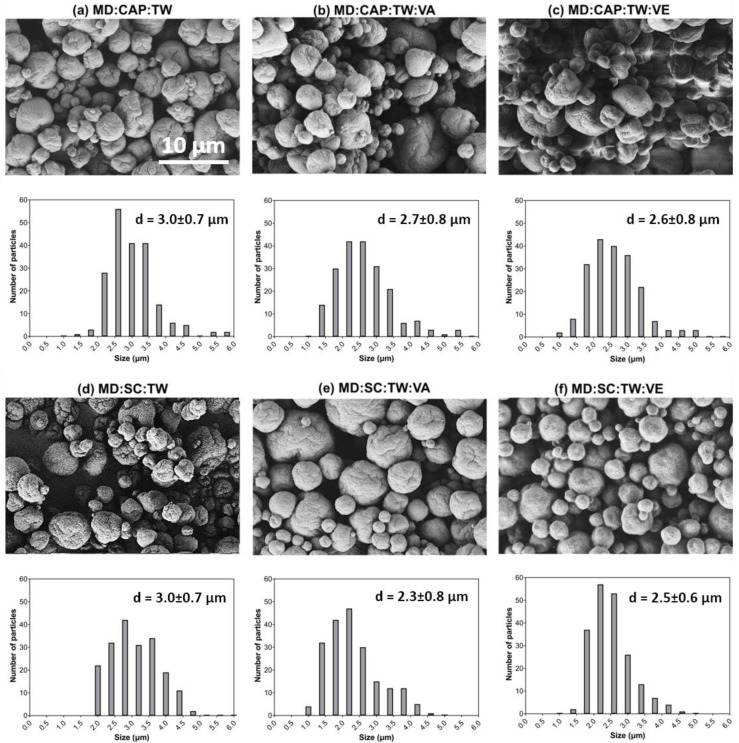
Scanning electron micrographs and histograms of particle size of vitamins encapsulated and controls. (**a**) MD:CAP:TW; (**b**) MD:CAP:TW:VA; (**c**) MD:CAP:TW:VE; (**d**) MD:SC:TW; (**e**) MD:SC:TW:VA (**f**) MD:SC:TW:VE.

The surface morphology of the encapsulated vitamin A and E was assessed by means of field-emission scanning electron microscopy (FE-SEM), which obtained images are shown in Figure 1.

In general, all the microcapsules revealed a semi-spherical shape, a rough surface and no evidence of holes or fissures, which suggests low permeability to gases and improved protection and retention of vitamins [25]. The control microcapsules have more surface dents than the encapsulated vitamin samples, which may be attributed to the shrinkage during the spray drying process [11,41,42]. As well, this phenomenon may be associated with the wall materials containing a high proportion of carbohydrates, in this case, MD [11]. Vitamin encapsulated samples exhibited less surface indentation and samples with MD:SC combination showed less agglomeration and a better-defined round shape. In general, the observed morphology is similar to that reported in the literature for microcapsules obtained with the same wall materials [25,41,43].

The particle size was also analyzed in terms of histograms, and the results are plotted in Figure 1. All the samples of encapsulated vitamin presented a wide mono-modal particle size distribution with a more frequent diameter in the range from 2 to 4 μm. The average particle size of the major population of particles was included in the histograms of Figure 1 as the mean value. The ANOVA analysis showed there were no significant differences between the different compositions (*p* < 0.05).

#### 2.2.2. Product Yield

The drying yield of the products is shown in Table 2. In general, the yields varied from 77.3% to 84.6%, including control microcapsules, and the average value was 81% ± 2%. According to Bhandari et al., product yield higher than 50% may indicate a successfully spray drying process [44]. The consideration of MD:CAP:TW and MD:SC:TW as encapsulating agents was, therefore, a good alternative to recover most of the initial mass at the end of the vitamin emulsion and spray-drying.

Several factors are involved in the product yield, such as the wall material selection, the vitamin-wall ratio and the drying conditions. In this regard, the majority presence of MD with low dextrose equivalence (DE) of 20 with a glass transition temperature (T_g_) of 141 °C [45,46], may increase the overall T_g_ of the mixture and, therefore, avoid the transition of the capsules from the glassy to the rubbery state and subsequent particle sticking to the chamber walls during drying at the outlet temperature of 74 °C [47,48,49,50,51]. In terms of vitamin-wall ratio, the selection of 1:6 (w:w) proportion may have furtherly contributed to preventing vitamin droplets from sticking on the chamber surface. Finally, it is important to remark that the drying process was carried out in a small surface area that promoted the heat and mass transfer, removing water more efficiently [45,49,52].

#### 2.2.3. Encapsulation Efficiency

The calculated encapsulation efficiency is gathered in Table 2. The higher efficiency value was found when MD:CAP:TW was used as an encapsulating agent. In particular, the encapsulation efficiency for vitamin A using MD:CAP:TW was 100% and that of vitamin E was 48%, respectively. However, the use of MD:SC:TW decreased encapsulation efficiency. Values lower than 30% were obtained both for vitamin A and E. This behavior could be explained by the feasible changes of the structure of sodium caseinate during ultrasonication, where proteins could be partially or totally denatured [53]. Furthermore, it was found that vitamin A was not totally distributed homogenously inside the matrix, which was furtherly assessed in the next sections.

#### 2.2.4. Moisture Content

The moisture content of the dried powders is gathered in Table 2. In general, results were in the range from 5.93% to 8.24% for MD:CAP:TW based microparticles and from 1.91% to 6.47% for MD:SC:TW based combinations. According to Tontul and Topuz, the moisture content of powder produced by spray-drying must be lower than 5%, allowing long-term product storage [45]. Given the absence of a dehumidification accessory in the inlet air of the spray drying process to control its relative humidity [49], values slightly above 5% were found [51]. Moreover, some studies revealed that the use of MD with a high dextrose equivalent (DE), as in this case (DE20), might contribute to increasing the sample hygroscopicity due to a great number of ramifications with hydrophilic groups. Thus, considering an average environmental relative humidity of 33%, samples may easily absorb moisture from the ambient air during powder handling after spray drying [38,54,55,56]. Therefore, the handling and storage of the powder are crucial processes that must be carefully carried out to avoid the humidity uptake.

#### 2.2.5. Porosity

The porosity is an important physical parameter for ascertaining the stability of food additive powders during storage [57]. For the porosity calculation, apparent (bulk) density and true particle density must be previously determined. Table 2 gathers the obtained density and porosity values (%) of vitamin encapsulated samples and controls. In general, values of bulk density and true particle density were similar regardless of the type of encapsulating agent or vitamin used (*p* < 0.05). The porosity values were in the range between 60% and 69%, in accordance with other spray-dried powders containing MD [57]. Although calculated porosity was high, they may not be considered as critical. It has been reported that values above 70% may result in significant oxygen diffusion and subsequently in the occurrence of undesirable oxidation reactions [57].

#### 2.2.6. Structural Analysis

**Figure 2:**
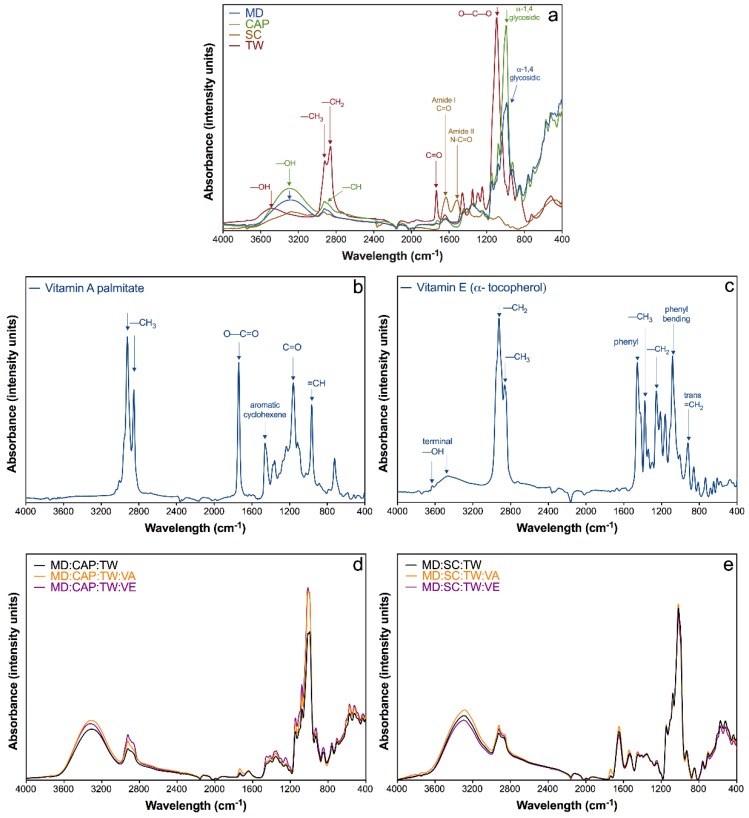
Fourier transform infrared spectroscopy (FTIR) spectra of (**a**) encapsulating agents—maltodextrin (MD), Capsul^®^ (CAP), sodium caseinate (SC)—and surfactant Tween 80 (TW); (**b**) Vitamin A (VA); and (**c**) Vitamin E (VE); and comparison of the vitamin encapsulation using (**d**) MD:CAP:TW and (**e**) MD:SC:TW matrices.

Fourier transform infrared spectroscopy (FTIR) analyses were performed in order to assess the chemical interactions between vitamins and encapsulating agents. First, all the bands associated with functional groups of each component were studied, as shown in Figure 2a for the encapsulating agents maltodextrin (MD), OSA-modified starch Capsul^®^ (CAP) and sodium caseinate (SC), including the surfactant Tween 80 (TW). In addition, the spectra of pure vitamins A and E are shown in Figure 2b,c, respectively. The powders of the encapsulated vitamins and controls are included in Figure 2d,e.

Both carbohydrates, MD and CAP, revealed a similar spectrum (Figure 2a). They present analogous characteristic absorption bands along with a common region from 1500–800 cm^−1^ known as “the fingerprint” of sugars. This region is characterized by an extensive overlapping of bands and vibrations, which are attributed to the C=O stretching and –COC–, –CHO and –CCH groups bending, and symmetrical –CH_2_– group deformations [58,59]. As well, strong bands appeared at 988 and 992 cm^−1^ in MD and CAP, respectively, which are attributed to the α-1,4 glycosidic linkages [58].

On the other hand, the SC infrared spectrum presented a wide band between 3600 and 2000 cm^−1^, which corresponds to the tension of the –NH group, characteristic of amino acids of isolated peptide chains [60]. According to Pereda et al., a smaller peak at 3000 cm^−1^ could be attributed to primary amines structure, being probably an overtone of amide II [61]. As well, it is possible to observe a –CH stretching region being assigned to –CH_2_– (2925 cm^−1^) and –CH_3_ (2970 cm^−1^) groups [61]. A second region between 1700 and 1500 cm^−1^ is due to amide I and II, common in proteins: The first peak at 1635 cm^−1^ corresponds to the stretching of the C=O group and the second, at 1512 cm^−1^, corresponds to symmetric stretching of N–C=O bonds. As well, a weak band at 1400 cm^−1^ is assigned to the O–C–O group, and finally, two bands at 1235 and 1075 cm^−1^ are ascribed to the C–O stretching in C‒OH bonds [60,61,62].

The spectrum of Tween 80 (TW) (Figure 2a) showed a broad band at 3498 cm^−1^ corresponding to –OH stretching vibrations [63,64]. Methyl groups originated a band at ~2900 cm^−1,^ and the asymmetric and symmetric stretching of –CH_2_ were confirmed at 2860 cm^−1^. The absorbance at 1734 and 1094 cm^−1^ were attributed to C=O and C–O–C stretching [63,64,65].

The spectrum of pure vitamin A (Figure 2b) showed the characteristic bands at 2922 and 2853 cm^−1^ of the free CH_3_ group stretching, 1739 cm^−1^ correlated to the O–C=O stretching, and various peaks from 1460 to 1160 cm^−1^ related to the –CO groups stretching [9,66,67]. A peak at ~1500 cm^−1^ could be attributed to the aromatic cyclohexene stretching [9,68]. All these peaks involve the hydroxyl, methyl and ester functional groups of vitamin A.

The pure vitamin E spectrum (Figure 2c) exhibited a wide band at 3473 cm^−1^ related to the terminal –OH group. Two bands at 2923 and 2864 cm^−1^ corresponded to asymmetric and symmetric stretching vibrations of –CH_2_– and –CH_3_, respectively [69,70]. The presence of a skeletal phenyl was indicated at 1457 cm^−1,^ and a peak at 1377 cm^−1^ was attributed to methyl symmetric bending. The band at 1262 cm^−1^ was characteristic of –CH_2_ groups, and peaks at 1080 and 919 cm^−1^ were assigned to plane bending of phenyl and trans =CH_2_ and C-O stretching [42,69,70]. All these peaks were related to the hydroxyl, methyl, phenyl, and ether functional groups of vitamin E.

In Figure 2d,e, the obtained spectra of all the powders were plotted. Both controls and samples with vitamins showed a strong band at ~3300 cm^−1^, which corresponds to the –OH group stretching. The presence of water may contribute to the higher intensity of this characteristic band, something common to find in a carbohydrate-water system due to the high hygroscopicity of MD, the main component of all the powders (~52%) [22,59,71]. The bands at ~2900 cm^−1^ represent the stretching vibration of –CH– bonds [71].

In general. the spectra for encapsulated vitamin samples showed similar bands than controls for both combinations of encapsulating agents (Figure 2d,e). However, the vitamin A microcapsules, both based in MD:CAP and MD:SC combinations, presented a weak band at ~1736 cm^−1^ characteristic of the pure vitamin A. This observation may indicate that vitamin A is present on the surface of the microcapsules. On the other hand, the vitamin E encapsulated samples revealed a spectrum without the characteristic peak of the pure vitamin, which may suggest that it was completely covered by the wall materials. Besides, it is important to note that any new peaks were found in the samples, which suggested that only physical interactions took place among the vitamin and the encapsulating agents and that no chemical reactions occurred between them.

#### 2.2.7. Thermal Properties

The extrusion process is one of the most extended processing methods in the fish feed industries. However, the decomposition of vitamins at high temperatures is one of the main drawbacks of this technology [17]. The evaluation of the thermal properties of the additives for fish pellets containing vitamins is therefore essential to determine their stability during processing. As well, the thermal analysis is a useful technique to assess the feasible interactions between the components of the prepared microcapsules.

**Figure 3:**
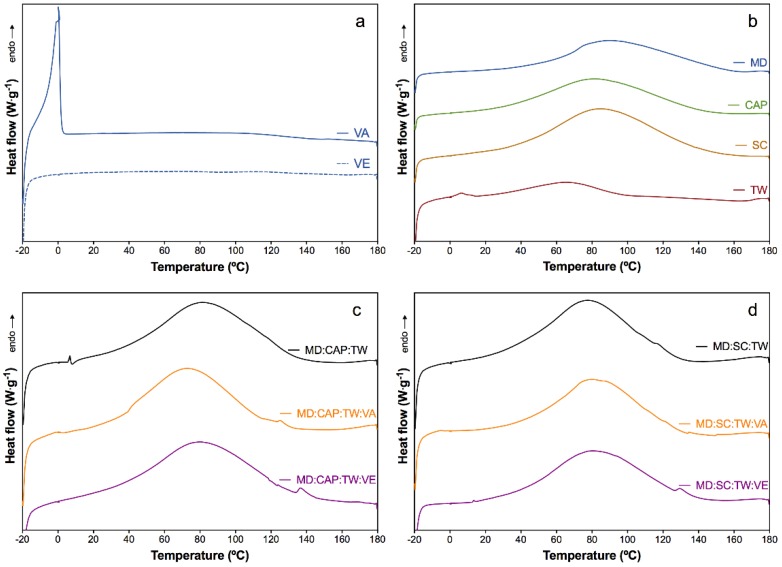
First heating differential scanning calorimetry (DSC) thermograms of (**a**) vitamin A (VA) and vitamin E (VE); (**b**) MD, CAP, SC, and TW components and comparison between encapsulated vitamins using (**c**) MD:CAP:TW and (**d**) MD:SC:TW wall polymer matrices.

The thermal properties were assessed by means of differential scanning calorimetry (DSC), in which thermograms of the first heating scan are plotted in Figure 3. The characteristic peak temperatures (T_p_) and enthalpies (∆h) are gathered in Table 3. Moreover, the calculated remnant water content in the microparticles was included.

Pure vitamin A (VA) revealed a sharp endotherm transition from −10 to 5 °C attributed to the melting of the crystallized oils, while vitamin E (VE) showed a flat thermogram (Figure 3a), according to the literature [72,73]. The maltodextrin (MD) and Capsul^®^ (CAP) are starch-derived materials, and their typical endothermic peak attributed to the gelatinization of starch, which depends on the heating rate and the origin and structure order of the starch [74]. For example, Borde et al., and Nurhadi et al. [75,76] showed the gelatinization peak value at 50 °C at a low heating rate for MD (3–5 °C·min^−1^), meanwhile, for a high heating rate of 20 °C·min^−1^ it was reported at 72 °C for MD and 60 °C for CAP [74]. In the present study, using a heating rate of 10 °C·min^−1^, the broad endothermic peaks attributed to gelatinization starch were observed at 90.4 °C and 81.4 °C for pure MD and pure CAP, respectively (Figure 3b). As well, the evaporation of free water molecules of the humidity content found in previous sections may simultaneously occur during these processes [77,78]. The sodium caseinate (SC) is a protein, which becomes generally denatured in the range from 50 to 80 °C, depending on their moisture content [53]. In this study, the denaturation process of SC was observed as an endothermic transition from 34 to 143 °C, with a peak value at 85.2 °C (Figure 3b). This high value and smooth endothermal peak were attributed to the low moisture content of the sample [53].

The Tween 80 (TW) showed two characteristic peaks at 6.3 °C and 66.4 °C [79,80]. Besides, its flashpoint was observed as an endothermic peak above 150 °C (~175 °C), as specified by the manufacturer (Figure 3b).

All the spray-dried samples showed a broad endothermic peak in the first heating scan around 100 °C (Figure 3c,d) that disappeared in the second heating scan. This observation can be correlated to the free water release [81,82]. In general, the samples containing MD:CAP:TW or MD:SC:TW showed the same thermal behavior regardless of the presence of vitamins. The slight differences in the enthalpies and temperature of the peaks between samples and controls are not representative and can be attributed to the minor differences in the remnant water content and the interaction within the different components. The calculated remnant water percentage moved between 5.5% and 6.7%, which is closely related to the moisture content shown in the previous section by gravimetric analysis. The presence of vitamin A and E resulted in a similar thermal behavior than the matrices. However, for samples containing vitamin A, a slight endothermic transition at ~0 °C, analogous to that plotted in Figure 3a. Therefore, this endothermic transition would corroborate the presence of vitamin A.

#### 2.2.8. Vitamin A Retention after One-Month Storage

The evaluation of the thermal properties permitted to assess the release of the vitamin in the samples after being stored during one month at 20 °C and 0% relative humidity (RH). In this regard, the ratio of the melting enthalpies associated with the vitamin in the sample and the pure vitamin may bring the proportion of remnant vitamin in the matrix [83,84].

**Figure 4:**
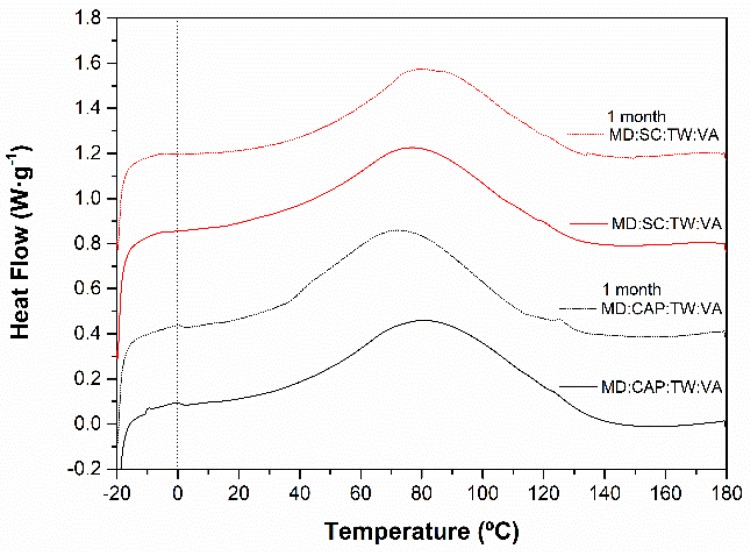
First heating DSC thermograms of MD:CAP:TW:VA and MD:SC:TW:VA powders after 1-month storage.

Given the small endothermic peak found for the powders containing vitamin A (VA) (MD:CAP:TW:VA and MD:SC:TW:VA), the stability after storage was assessed in these samples. The original calorimetric thermograms, along with those obtained after 1-month storage, are plotted in Figure 4.

The results revealed almost identic vitamin A melting enthalpy after storage, and therefore, the percentage of released vitamin A during storage was negligible regardless of the encapsulating agent. This observation was considered after comparison with the calculated initial encapsulation efficiency for both encapsulating agents (Table 4). Altogether, the retention of vitamin A into the microcapsules after 1-month storage was demonstrated.

#### 2.2.9. Thermo-Oxidative and Thermal Stability

**Figure 5:**
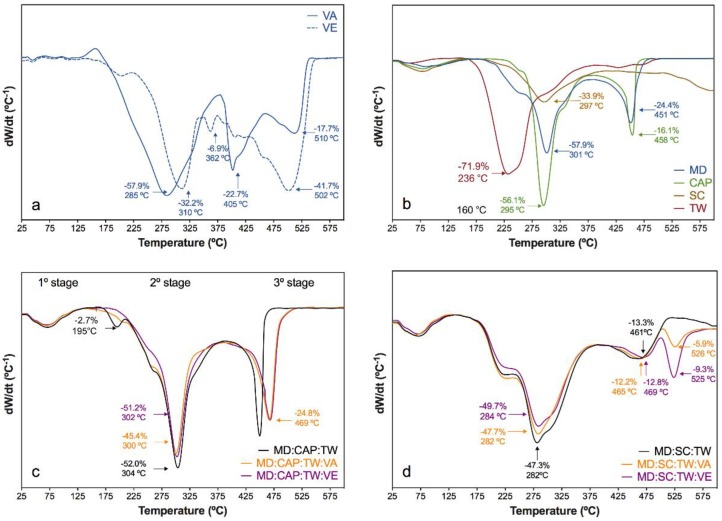
DTGA curves under oxidative atmosphere of (**a**) vitamin A (VA) and vitamin E (VE); (**b**) MD, CAP, SC, and TW components; and comparison of the vitamin encapsulation using (**c**) MD:CAP:TW and (**d**) MD:SC:TW matrices.

**Figure 6:**
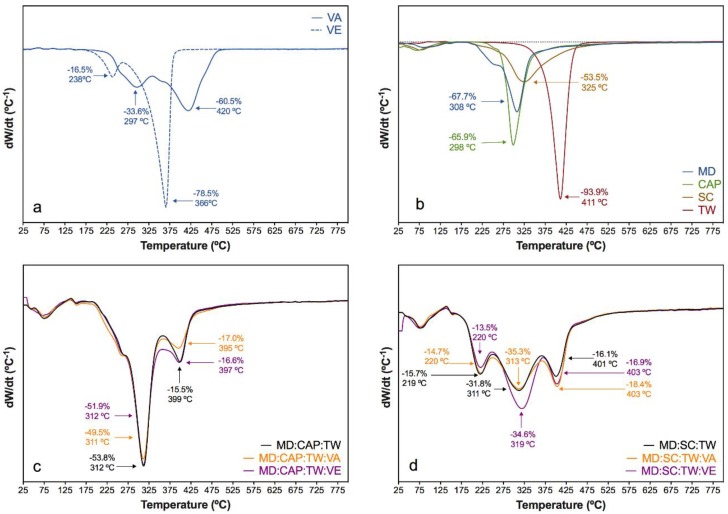
DTGA curves under an inert atmosphere of (**a**) vitamin A (VA) and vitamin E (VE); (**b**) MD, CAP, SC, and TW components; and comparison of the vitamin encapsulation using (**c**) MD:CAP:TW and (**d**) MD:SC:TW matrices.

The thermogravimetric analysis (TGA) permits to evaluate the thermal stability of the prepared microcapsules under different atmospheres. In this study, oxidative and inert atmospheres were considered, in which derivative decomposition profiles (DTG) are shown in Figure 5 and Figure 6, respectively. The peak temperatures and percentages of mass loss of the different stages are included in the plots.

Regarding the thermo-oxidative stability, both vitamin A and E revealed a main decomposition stage from 150 °C to 350 °C, where major weight loss occurred (Figure 5a), followed by subsequent degradation in two stages for the VA and one stage for the VE from 380 °C onwards [67]. The encapsulating agents MD, CAP, and SC (Figure 5b) presented dehydration from 25 °C to 150 °C with a mass loss of 5%–6%, in which free water was released from the microparticles, in line with the results of previous sections [85,86,87]. Then, the main decomposition stage occurred at 301 °C for MD, at 295 °C for CAP, and at 297 °C for SC [86,88]. Finally, the volatilization of MD and CAP occurred around 450 °C. The TW revealed the main decomposition stage at 236 °C, with a mass loss of 72%, which progressively decomposed as a function of temperature.

Both the MD:CAP:TW and the MD:SC:TW controls revealed the combination of the previously described processes. In particular, the MD:CAP:TW microcapsules (Figure 5c) showed a shoulder at 250 °C, to which MD and TW contributed, followed by the main decomposition stage around 300 °C. The MD:SC:TW microcapsules (Figure 5d) revealed a wider main decomposition stage from 175 to 400 °C due to the SC decomposition. Afterward, the volatilization of MD was perceived from 470 °C onwards in both compositions.

For the vitamin encapsulated samples based in MD:CAP:TW, an almost identic profile to that of the control was found. However, the peak at 450 °C moved to 469 °C when VA and VE were incorporated, representing an intermediate behavior between the control and vitamins. Moreover, the mass loss during this stage increased from 20% for the control to around 25% for the vitamin encapsulated samples. The presence of both vitamin A and E was, therefore, corroborated.

The thermo-oxidative decomposition of vitamin encapsulated in MD:SC:TW was also similar to that of the control. Nevertheless, a significant mew decomposition step occurred around 525 °C. This peak is related to the degradation of vitamin-encapsulation agent complexes, and a mass loss contribution of 8.5% for VA and 12.3% for VE encapsulated samples was found. Although a similar peak was observed in the DTGA curves of the pure vitamins, the higher temperature was perceived in this stage for the vitamin-containing microcapsules. Therefore, the interaction between vitamins and encapsulating agents was expected.

Under an inert atmosphere, a different decomposition behavior was found. The thermal degradation involved more concise stages, with sharper peaks in the DTGA curves. The pure vitamins A and E showed a two-stage behavior (Figure 6a). For VA, a first shoulder was perceived at 297 °C, and the main decomposition was found at 420 °C. The VE revealed a small shoulder at 238 °C, followed by the main stage at 366 °C [67,89]. The encapsulating agents (Figure 6b) revealed a first dehydration stage until 130 °C, followed by a one-step thermal decomposition at 308 °C for MD, 298 °C for CAP, and 325 °C for SC. In particular, the MD revealed a slight shoulder at 240 °C [62,88,90]. The thermal decomposition of TW occurred in a single concise stage at 411 °C.

The DTGA curves of the control and vitamin-encapsulated samples comprised the dehydration reaction until 130 °C along with the degradation steps of the pure components. For the MD:CAP:TW-based microparticles (Figure 6c), the main decomposition occurred around 310 °C. Additionally, a decomposition stage around 400 °C was found, unperceivable for the pure components, which involved the thermal decomposition of previously formed by-products. Although VA decomposition revealed a peak at 420 °C and could be the responsible of the latter decomposition, the presence of this peak in samples containing VE suggests that it was due to the decomposition of char. Therefore, an interaction between the encapsulation agents during the thermogravimetric assay was expected.

However, the characteristic peaks of vitamins may have been overlapped by the previously described thermo-decomposition stages. Only for the VE-based microcapsules, the contribution of vitamin decomposition may be intuited in between the above-described peaks, around 350 °C. For the microcapsules based in MD:SC:TW (Figure 6d), dehydration was followed by three stages, in which that of SC at 311 °C gained importance. The previously described stage at 400 °C due to char decomposition was found again.

When compared the thermal stability under the oxidative and inert atmospheres, dissimilar behavior was perceived due to the oxidative and pyrolytic processes, respectively. However, the thermogravimetric analysis revealed that the encapsulation of vitamins did not alter their thermal and thermo-oxidative stability. In this sense, all the prepared capsules are suitable candidates for being used as nutritional additives, given its stability up to 170 °C regardless of the inert and oxidative atmospheres, and above the temperatures commonly used during the extrusion processes [91].

## 3. Materials and Methods

### 3.1. Materials

Vitamin A (VA) Palmitate was provided by BASF (Chile), and vitamin E (dl-α-Tocopherol) (VE) was purchased from Sigma-Aldrich (USA). Tween 80 (TW) (Winkler, Chile) was used as an emulsifier. The encapsulating agents, maltodextrin (DE 20) (MD) and OSA-modified starch (Capsul^®^) (CAP) were provided by Quimatic (Chile) [92]. The sodium caseinate (SC) was provided by Blumos (Chile).

### 3.2. Sample Preparation

#### 3.2.1. Nanoemulsions

**Figure 7:**
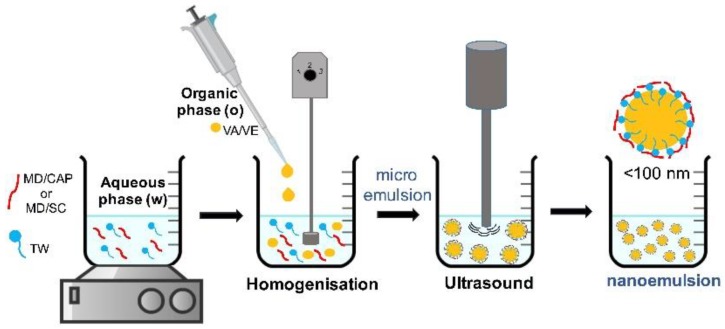
Nanoemulsion preparation sequence.

The nanoemulsion preparation was carried out according to the Figure 7. The aqueous phase was prepared by adding the encapsulating agents to distilled water. Two different combinations were used, in both cases at a 70:30% w/w such as shown the Table 4. Maltodextrin (MD) was combined with Capsul^®^ (CAP) and sodium caseinate (SC) separately. Mixtures were stirred magnetically at 650 rpm and 50 °C for 90 min. After that, Tween 80 (TW) (1% w/w) was added to the aqueous phase and the solution was stirred for a further 30 min. Then, the organic phase (1% w/w), composed by a lipophilic vitamin (A or E) was incorporated drop by drop into the aqueous phase, while it was homogenized using a T25 digital Ultra-Turrax^®^ (IKA, Staufen, Germany) operating at 10^4^ rpm for 15 min in order to obtain a coarse emulsion of oil in water (o/w). Finally, in order to obtain nanoemulsions, the coarse emulsion was homogenized by ultrasound treatment with a stainless-steel ultrasound probe (13 mm diameter; QSonica, Newtown, CT, USA). Ultrasound homogenization was performed at 80% of the amplitude and 20 kHz of frequency for during 15 min. For comparison purposes, control samples for the plain encapsulating agents and surfactants were prepared.

#### 3.2.2. Spray Drying Conditions

The spray-drying was carried out using a Mini Spray Dryer B-290 equipment (BÜCHI Labortechnik AG, Flawil, Switzerland) with a standard 0.7 mm nozzle. All nanoemulsions were prepared with the following procedure. Spray-drying was performed before 24 h of nanoemulsion preparation, given its previously determined physical stability. The nanoemulsions were spray-dried with a liquid feed volumetric flow rate of 2 mL·min^−1^, nozzle air flow-rate of 1.052 m^3^·h^−1^ and aspiration of 80% (32 m^3^·h^−1^). The inlet and outlet temperatures were 120 ± 1 °C and 74 ± 1 °C, respectively. The spray-dried powder samples were collected, kept in amber glass bottles and stored in a desiccator at 23 ± 1 °C until further analyses.

### 3.3. Emulsion Characterization by Droplet Size and Polydispersity Index

The average particle size and its distribution were measured by dynamic light scattering using a Zetasizer Nano-ZS90 instrument (Malvern Instruments, Worcestershire, UK). The refractive indexes were determined using a refractometer HI 96801 (Hanna Instruments, Romania). Values for the disperse phase were 1.51 and 1.50 for vitamins A and E, respectively. For the dispersant phase (water-encapsulating agents-Tween 80), values were 1.34 and 1.33 for MD in combination with CAP and SC, respectively. The nanoemulsions were diluted in deionized water to optimize the analysis. Measurements were made in triplicate with 10 runs each at 25 °C. The droplet size and the size distribution were expressed by the cumulants mean diameter (Z-average) and the polydispersity index (PDI), respectively, which were calculated by Zetasizer Software v7.10 (Malvern Instruments, Worcestershire, UK).

### 3.4. Powder Characterization

#### 3.4.1. Product Yield and Encapsulation Efficiency

The spray drying yield is defined as the fraction of solid material recollected after the process in comparison to the initially amount of solid material incorporated, expressed as a percentage [93]. The powder yield was calculated according to Equation (1).

(1) $$Product\;yield\;\left(\%\right)=\frac{Final\;mass\;of\;dried\;capsules\;\left(g\right)}{Initial\;mass\;of\;solid\;material\;\left(g\right)}\times 100$$

The encapsulation efficiency was calculated by means of Equation (2). For vitamin surface content, 5 mL of hexane was added to 400 mg of encapsulated vitamin (A and E) powders, followed by a vortex agitation during 30 s and centrifugation at 5000 rpm for 5 min [41]. Once the phases were separated, an aliquot of 1 mL was extracted from the supernatant to measure the UV light absorbance in a Multiscan GO Microplate Reader (Thermo Fischer Scientific, USA). The wavelength for vitamin A and E was 271 nm and 298 nm, respectively. All the measurements were made in triplicates.

(2) $$Encapsulation\;efficiency\;\left(\%\right)=\frac{Total\;vitamin\;content\left(g\right)-Vitamin\;on\;surface\;\left(g\right)}{Total\;vitamin\;content\;\left(g\right)}\times 100$$

#### 3.4.2. Moisture Content

The moisture content was assessed according to Association of Official Agricultural Chemists (AOAC) (2000) [38] by gravimetric measurements into a drying oven with forced air circulation (Wiseven Daihan WOF-105, Seoul, Korea) at 105 °C until constant mass (30 h). The results were calculated as a dry weight basis (%db, g water/100 g dried sample) in triplicates. Moisture content analyses were carried out in the same batch, once all the formulations were prepared. In the meantime, the prepared microcapsules were saved into threaded glass vials, all of them stored into a desiccator at 23 ± 1 °C.

#### 3.4.3. Porosity

The porosity of the samples was calculated using the relationship between the bulk density and true density, according to Equation (3). The bulk density was measured using a 10 mL graduated cylinder filled with the sample and weighted. The true density was measured using an MVP 1305 pycnometer (Micrometrics Instrument Corp., Norcross, GA, USA). All the measurements were made in triplicates.

(3) $$Porosity\;\left(\%\right)=1-\left(\frac{bulk\;density\;\left(g\cdot cm^{-1}\right)}{true\;density\;\left(g\cdot cm^{-1}\right)}\right)\times 100$$

#### 3.4.4. Fourier Transform Infrared Spectroscopy (FTIR)

Microcapsules were characterized by Fourier transform infrared spectroscopy (FTIR) to confirm the absence of chemical interactions between vitamins and polymer and to assess the formation of the microparticles. The FTIR spectra were recorded in a Thermo Nicolet 5700 FT-IR spectrometer equipped with an attenuated total reflectance unit (ATR) (Thermo Electron Corporation, Madison, WI, USA). The spectra were collected in triplicate in the region between 4000 cm^−1^ and 400 cm^−1^ with a resolution of 4 cm^−1^ and 64 accumulations. A previous air-background correction was considered. The spectral analysis was performed by means of the Omnic^TM^ v7.0 software.

#### 3.4.5. Differential Scanning Calorimetry (DSC)

On the one hand, the thermal properties of the powders were evaluated by means of differential scanning calorimetry (DSC) in a Mettler Toledo DSC 820^e^ equipment (Columbus, OH., USA) previously calibrated following the procedure of In and Zn standards. The samples, with a mass of about 4 mg, were loaded in 40 μL aluminum pans, hermetically sealed and analyzed between −20 °C and 180 °C with a heating rate of 10 °C·min^−1^. All the experiments were run under N_2_ inert atmosphere at 50 mL·min^−1^. The results were analyzed using the STARe 15.0 software (Mettler Toledo, Columbus, OH, USA). The temperatures for the different thermal transitions were calculated as the peak values. As well, the variation of enthalpy (Δh) of the transitions was defined as the area under the peak. All the samples were characterized at least in triplicates and the averages of temperatures and enthalpies were reported as representative values.

The remnant water content was calculated from the water release enthalpy (Δh) according to Equation (4), considering a water evaporation enthalpy of 2418 J·g^−1^ [94].

(4) $$\%\;water\;content\;\left(\%w\right)=\frac{\Delta h_{w}}{\Delta h_{w0}}\times 100$$

On the other hand, the differential scanning calorimetry technique may allow to measure the percentage of the vitamin released after storage indirectly. In this study, the percentage of vitamin A was calculated following the Equation (5), as reported by Ponce-Cevallos et al. [83], where ∆h_A_ is the melting enthalpy of vitamin A in the capsules and ∆h_0_ is the heat of melting of pure vitamin A (43.2 J·g^−1^).

(5) $$\%\;vitamin\;released\;\left(\%v\right)=\frac{\Delta h_{A}}{\Delta h_{A0}}\times 100$$

#### 3.4.6. Thermogravimetric Analysis (TGA)

The thermo-oxidative decomposition profiles were obtained by means of a Mettler-Toledo TGA/SDTA 851^e^ thermogravimetric analyzer (TGA) (Columbus, OH., USA). The samples, with a mass of about 4 mg were introduced into TGA Mettler-Toledo perforated alumina crucibles, with a capacity of 70 μL. Then, they were analyzed from 25 °C to 600 °C with a heating rate of 10 °C·min^−1^, under an oxidative or inert atmosphere of O_2_ and Ar, respectively, at a flow rate of 50 mL·min^−1^. The experiments were performed in triplicates to ensure reproducibility. The results were evaluated using the STARe 15.0 software (Mettler Toledo, Columbus, USA).

#### 3.4.7. Particle Morphology by Field-emission Scanning Electron Microscopy (FE-SEM)

The surface morphology of the microcapsules was analyzed by means of a Zeiss Ultra 55 field emission scanning electron microscope (FE-SEM) (Oberkochen, Germany). The powders were placed onto conductive tape on metal studs and sputter-coated with a platinum layer during 10 s using a Leica EM MED020 sputter coater. The FE-SEM images were taken at 22 °C with a 1 kV voltage. The particle dimensions were measured from the scanning electron microscope images (5000×) at random locations (*n* = 200) using the ImageJ^®^ software v1.52a (National Institutes of Health, USA).

### 3.5. Statistical Analysis

All the measurements were performed in triplicates and results were reported as mean values and their corresponding standard deviations. The data were assessed by analysis of variance (ANOVA) with a significance level of 0.05 (*p* < 0.05). Multiple comparison tests were performed using the Bonferroni-Dunn method when the Bartlett’s test resulted in significance between variances (α = 0.05). All the statistical analysis was performed using GraphPad Prism software v7 (GraphPad Software, CA, USA).

## 4. Conclusions

The emulsification method combined with spray-drying was appropriate to encapsulate vitamin A and E using Tween 80 as an emulsifier in combination with maltodextrin/Capsul^®^ and maltodextrin/sodium caseinate as encapsulating agents.

The obtained nanoemulsions were stable, and the drying procedure was effective in obtaining microcapsules with a product yield above 75%, low moisture content, and tolerable porosity, highly required in the feed industry. Although slightly lower encapsulation efficiency was found with maltodextrin/sodium caseinate, the use of maltodextrin/Capsul^®^ resulted in higher efficiency.

The oxidation of vitamins during storage and conventional processing procedures such as extrusion in the feed industry involves a serious concern for pellet preparation. However, the stability after 1-month storage in low humidity conditions along with the thermal and thermo-oxidative stability of the vitamin A and E microcapsules with maltodextrin/sodium caseinate and maltodextrin/Capsul^®^ up to 170 °C, postulates the proposed system to avoid such described issues.

Altogether, the proposed encapsulation methodology is, therefore, a feasible alternative for the stabilization of vitamin A and E and protection against oxidation processes in the feed manufacturing industry.

## Figures and Tables

**Table 1:** Mean particle size and polydispersity index of Vitamin A (VA) and E (VE) nanoemulsion based on maltodextrin in combination with Capsul^®^ (CAP) or sodium caseinate (SC) and Tween 80 (TW).

Sample	Z-Average Size (nm)	PDI
MD:CAP:TW:VA	102.5 ± 1.3 ^a^	0.473 ± 0.091 ^a^
MD:CAP:TW:VE	75.1 ± 0.1 ^b^	0.286 ± 0.050 ^b^
MD:SC:TW:VA	100.4 ± 0.2 ^a^	0.431 ± 0.009 ^a^
MD:SC:TW:VE	80.1 ± 2.7 ^c^	0.263 ± 0.004 ^b^

^a,b,c^ Different letters in the same column indicate significant differences between samples (*p* < 0.05) analyzed using multiple comparison of Bonferroni-Dunn test.

**Table 2:** Product yield, encapsulation efficiency, moisture content, bulk density, and particle (true) density and porosity of microcapsules. MD: Maltodextrin, CAP: Capsul^®^, SC: Sodium caseinate and TW: Tween 80.

Sample	Product Yield (%)	Encapsulation Efficiency (%)	Moisture (%db)	Bulk Density (g·cm^−3^)	Particle Density (g·cm^−3^)	Porosity (%)
MD:CAP:TW	80.3 ± 1.2 ^ab^	-	8.1 ± 1.0 ^b^	0.44 ± 0.01 ^a^	1.14 ± 0.01 ^a^	62 ^ab^
MD:CAP:TW:VA	81.3 ± 2.0 ^abc^	100 ± 5	5.9 ± 2.2 ^ab^	0.40 ± 0.01 ^a^	1.12 ± 0.02 ^a^	64 ^a^
MD:CAP:TW:VE	79.7 ± 0.7 ^ab^	48 ± 3	8.2 ± 1.2 ^b^	0.37 ± 0.01 ^a^	1.06 ± 0.01 ^b^	65 ^a^
MD:SC:TW	77.3 ± 1.6 ^a^	-	6.5 ± 1.7 ^b^	0.44 ± 0.05 ^a^	1.09 ± 0.01 ^ab^	60 ^b^
MD:SC:TW:VA	83.1 ± 1.4 ^bc^	23 ± 4	1.9 ± 0.8 ^a^	0.35 ± 0.04 ^a^	1.25 ± 0.21 ^ab^	69 ^c^
MD:SC:TW:VE	84.6 ± 0.6 ^c^	29 ± 5	4.4 ± 1.5 ^ab^	0.40 ± 0.03 ^a^	1.10 ± 0.02 ^ab^	64 ^a^

^a,b,c^ Different letters in the same column indicate significant differences between samples (*p* < 0.05) analyzed using multiple comparisons of Bonferroni-Dunn test.

**Table 3:** Peak temperature (T_p_) and enthalpy values (Δh) of the main endothermic events in the first heating scan.

Sample	T_p1_ (°C)	Δh_1_ (J·g^−1^)	T_p2_ (°C)	Δh_2_ (J·g^−1^)	Water Content (%)
Vitamin A (VA)	~0	43.2	-	-	-
Vitamin E (VE)	-	-	-	-	-
MD:CAP:TW	-	-	82.1	155.6	6.4
MD:CAP:TW:VA	~0	0.4	81.4	157.3	6.5
MD:CAP:TW:VE	-	-	80.4	158.6	6.5
MD:SC:TW	-	-	77.2	161.2	6.6
MD:SC:TW:VA	~0	0.2	77.7	133.6	5.5
MD:SC:TW:VE	-	-	76.5	163.9	6.7

**Table 4:** Summary of the formulations achieved. Components: Maltodextrin (MD), Capsul^®^ (CAP), sodium caseinate (SC), Tween 80 (TW), Vitamin A (VA) and E (VE).

Samples	Wall Polymer Blends	Vitamin	Emulsifier
MD:CAP:TW:VA	MD:CAP (70:30 % w/w)	VA (1% w/w)	TW (1% w/w)
MD:CAP:TW:VE		VE (1% w/w)	
MD:SC:TW:VA	MD:SC (70:30 % w/w)	VA (1% w/w)	
MD:SC:TW:VE		VE (1% w/w)	
